# Carbon Monoxide Diffusion Capacity as a Severity Marker in Pulmonary Hypertension

**DOI:** 10.3390/jcm11010132

**Published:** 2021-12-27

**Authors:** Eleni Diamanti, Vasiliki Karava, Patrick Yerly, John David Aubert

**Affiliations:** 1 Division of Pulmonology, Lausanne University Hospital, University of Lausanne, CH-1011 Lausanne, Switzerland; eleni.diamanti@h-fr.ch; 2 1st Department of Pediatrics, Aristotle University of Thessaloniki, 541 24 Thessaloniki, Greece; vasilikikarava@hotmail.fr; 3 Division of Cardiology, Lausanne University Hospital, University of Lausanne, CH-1011 Lausanne, Switzerland; patrick.yerly@chuv.ch

**Keywords:** diffusion capacity, pulmonary hypertension, risk stratification, transplant-free survival

## Abstract

Carbon monoxide diffusion capacity (DLCO) is negatively associated with patient survival in idiopathic pulmonary hypertension (PH), but is not included in the risk stratification score proposed by the 2015 European guidelines. Since 2015, several new stratification scores based on a 3- or 4-severity scale have been explored. This retrospective cohort single-center study sought to investigate the association between DLCO and PH severity and survival. We included 85 treatment-naive patients with precapillary PH and DLCO measurement at diagnosis. DLCO status, based on lower and upper quartiles ranges, was added to a 3- and a 4-strata modified-risk assessment. DLCO was strongly associated with transplant-free survival (HR 0.939, 95% CI: 0.908–0.971, *p* < 0.001). In the intermediate and high-risk categories, DLCO was associated with transplant-free survival, irrespective of the risk category (HR 0.934, 95% CI: 0.880–0.980, *p* = 0.005). The correlation between modified-risk category and transplant-free survival was significant (HR 4.60, 95% CI: 1.294–16.352, *p* = 0.018). Based on the Akaike information criterion (AIC) levels, the 3- and 4-strata modified-risk stratification fits our results better than the conventional stratification. Low DLCO is associated with patient transplant-free survival, independently of the risk category. Inclusion of DLCO into a PH risk stratification score seems promising and needs further investigation.

## 1. Introduction

Pulmonary hypertension (PH) is a hemodynamic condition that results in high pulmonary arterial pressure (PAP) [1,2], as diagnosed by right heart catheterization (RHC), which is the established gold standard examination [3]. If left untreated, PH leads to right ventricular failure, and ultimately to death [4]. According to the 2015 European Society of Cardiology/European Respiratory Society (ESC/ERS) and 2018 World Symposium guidelines [2,5], PH is classified in five distinct diagnostic groups: Group 1 refers to pulmonary arterial hypertension (PAH), Group 2 to PH with left heart disease, Group 3 to PH with lung diseases or hypoxia, Group 4 to chronic thromboembolic PH (CTEPH) or other pulmonary artery obstructions, and Group 5 to PH with unclear or multifactorial mechanisms. The disease severity in PH patients comprising mainly those pertaining to Groups 1 and 4 is usually determined according to 2015 ESC/ERS risk assessment strategy. Patients stratified into high-risk category exhibit an estimated one-year risk of death >10% [2]. Presently, regular comprehensive risk assessment involving multiple variables, notably clinical, functional, and laboratory parameters, is strongly recommended for optimal initial and follow-up evaluations, estimation of long-term prognosis, guidance of treatment strategy, and monitoring of treatment response in PH patients [6,7,8].

Single-breath carbon monoxide diffusing capacity (DLCO) alteration can result from alveolo-capillary membrane damage, pulmonary micro-vessel area reduction, alveolar capillary volume, lung perfusion, smoking habits, potential intrapulmonary or intracardiac shunts or, finally, from low hemoglobin concentration [9,10]. To eliminate this last etiology, DLCO is corrected using standard hemoglobin content (DLCOc). A DLCO value < 50% predicted is strongly suggestive of pulmonary vascular disease or parenchymal disorder [11]. DLCO is a well-established pulmonary function test for the diagnostic evaluation of patients with suspected PH [12]. In systemic sclerosis patients, predicted DLCO < 60% was shown strongly associated to PAH in mildly symptomatic patients [13], while DLCO/alveolar volume (VA) < 60% predicted eventual PH within a 36-month time interval [14,15]. In addition, there is strong evidence for DLCO to be a useful prognostic and follow-up marker [16,17]. Indeed, DLCO was included in the REVEAL (Registry to Evaluate Early and Long-term PAH Disease Management) score 1.0 for risk-calculator of 1-year survival in patients with either PAH or CTEPH [18,19]. In contrast, DLCO was not evaluated in the French registry [20], nor was it included in 2015 ERS/ESC disease activity variable set [2]. Moreover, the DLCO threshold level at 32%, which was used for risk stratification in the initial REVEAL score version, was upgraded to ≤40% in its most recent version (REVEAL 2.0) [7]. Therefore, the significance of DLCO and its cut-off value for PH risk assessment remain controversial.

Given this context, this study was aimed to investigate the relationship between DLCO at diagnosis and transplant-free survival in PH patients, as well as to define the DLCO threshold level associated with worse patient prognosis. Secondary endpoints were to assess: (1) correlation between DLCO and other parameters used for risk assessment; (2) DLCO’s added value in currently established risk assessment scores, including the one derived from COMPERA (Comparative, Prospective Registry of Newly Initiated Therapies for PH) registry and described by Hoeper et al. [21].

## 2. Materials and Methods

We conducted a retrospective cohort study based on the medical records of adult PH-diagnosed patients followed-up in our PH clinic from January 2004 to December 2017. Inclusion criteria were as follows: (1) RHC confirming precapillary PH in treatment-naive patients; (2) DLCO measurements available before treatment initiation and within 8 months from RHC. Based on 2015 guidelines [2], precapillary PH was defined by a mean PAP > 25mmHg, pulmonary artery wedge pressure (PAWP) ≤ 15mmHg, and pulmonary vascular resistance (PVR) > 3 wood units (WU). The new hemodynamic definition of pre-capillary PH by a mean PAP > 20mmHg, as proposed at the 6th World Symposium on PH [5], was only published after the time of diagnosis and was, therefore, not applied in the current study. For text simplicity, we are using the DLCO acronym when discussing physio-pathological mechanisms and DLCOc when referring to current study data.

DLCOc, transfer coefficient for carbon monoxide (KCO), and VA measurements were performed using a single-breath diffusing capacity technique, in line with the 1993 ECCS/ERS equation [22], mostly at our institution, whereas a minority of pulmonary function tests was effectuated at the referral site. Correction for hemoglobin level was performed for both DLCOc and KCO levels, as recommended by the 2017 American Thoracic Society (ATS)/ERS standards [23]. Likewise, pulmonary function tests were in line with ATS acceptability and repeatability criteria [24]. In addition, we analysed the transplant-free survival with Kaplan–Meier curves according to several DLCOc cut-off values from 30 to 70% by 5% increment. The DLCO value with the best *p* value on log-rank test was chosen as cut-off.

We collected demographic, clinical, and laboratory data at diagnosis including gender, age, New York Heart Association (NYHA) functional class, serum N-terminal pro-brain natriuretic peptide (NT-proBNP) and 6-min-walk test (6MWT) distance. The following pertinent data were extracted from initial RHC: systolic pulmonary arterial pressure (sPAP), mean pulmonary arterial pressure (mPAP), cardiac output (CO), right atrial pressure (RAP), cardiac index (CI), pulmonary vascular resistance (PVR), and mixed venous oxygen saturation in pulmonary artery (SvO2). Statistical analysis was carried out for the variables with missing data <15%.

Risk stratification assessment was performed using the abbreviated version of risk assessment score, which had been validated and published by the COMPERA registry and Hoeper et al. study [21]. The six parameters including functional class NYHA, 6-min-walk-test (MWT) distance, NT-proBNP, RAP, CI, and SvO2 were graded for each patient from 1 to 3 (1: low-risk; 2: intermediate-risk; 3: high-risk) according to the proposed cut-off values [21]. The sum of these grades was then divided by the number of available parameters and rounded to the next integer in order to define the risk group for each patient. Patients with less than five parameters or without an available 6WMT were excluded from the risk stratification assessment.

We included DLCOc as additional parameter into the Hoeper’s et al. model, and then divided the patients into three groups, based on interquartile, lower (25th), and upper quarter (75th) of DLCOc ranges. The first and the third quartiles, expressed as DLCO % of predicted values, were rounded down to the nearest number multiple of 5. Patients exhibiting DLCOc <1st quartile, ≥1st quartile and <3rd quartile, and ≥3rd quartile were classified into low, intermediate, and high-risk categories, respectively. We then recalculated the risk group for each patient, which was defined as a modified-risk assessment. To do so, we proceeded like in the original risk assessment: we graded DLCO from 1 to 3 (1: low-risk; 2: intermediate-risk; 3: high-risk) according the quartile, added it to the other parameters of Hoeper’s et al. score and divided the sum by the number of available parameters.

Finally, we performed risk stratification based on the refined 4-strata risk assessment model, as published recently by COMPERA 2.0 group [25]. The three parameters, functional class NYHA, 6-MWT distance and NT-proBNP, were graded from 1 to 4 (1: low-risk, 2: intermediate-low risk, 3: intermediate-high risk and 4: high risk); the sum was divided by 3, rounded to the next integer, which defined the 4-strata risk group for each patient. Then, based on lower quartile (25th), median (50th) and upper quartile (75th) of DLCOc ranges, we divided the patients into four groups. Patients exhibiting DLCOc <1st quartile, ≥1st quartile and <2rd quartile, ≥2rd quartile and <3th quartile, and ≥3th quartile were graded from 1 to 4. Then we added DLCOc to the other parameters of COMPERA 2.0 4-strata risk, divided the sum by four and, thus, calculated the modified-4-strata risk assessment.

All statistical analyses were performed using SPSS software 22.0 (IBM, New York, NY, USA). Continuous variables were expressed as median values and ranges. Correlations between DLCOc and PH disease severity parameters were assessed using univariate and multiple linear regression analyses. Mann–Whitney test was employed to compare the study parameter distribution between patients exhibiting either DLCOc > 55% or ≤55%. This DLCOc cut-off was the number, multiple of five, nearest to the median, which was significantly associated with survival. Differences in DLCOc distribution among the risk stratification categories were studied based on the Kruskal–Wallis test. Kaplan–Meier survival analysis, as well as univariate and multiple Cox regression analyses were used to determine transplant-free survival risk factors. In survival analysis, the event endpoint was defined as time of patient death or lung transplantation, while the censored endpoint referred to the time of last patient visit. Cox regression models were compared based on the Akaike information criterion (AIC) values [26]. Logistic regression analysis was employed for identifying 3- and 5-year survival risk factors. Finally, receiver operating characteristic (ROC) curves were applied and area under the curve (AUC) were calculated to compare the risk stratification scores. The Δ between the 2 AUC models was measured. Results were considered statistically significant for *p* value < 0.05.

Oral or written consent was obtained from all participants. The study was approved by the local ethical committee (CER-VD No 2019-02475).

## 3. Results

**Figure 1:**
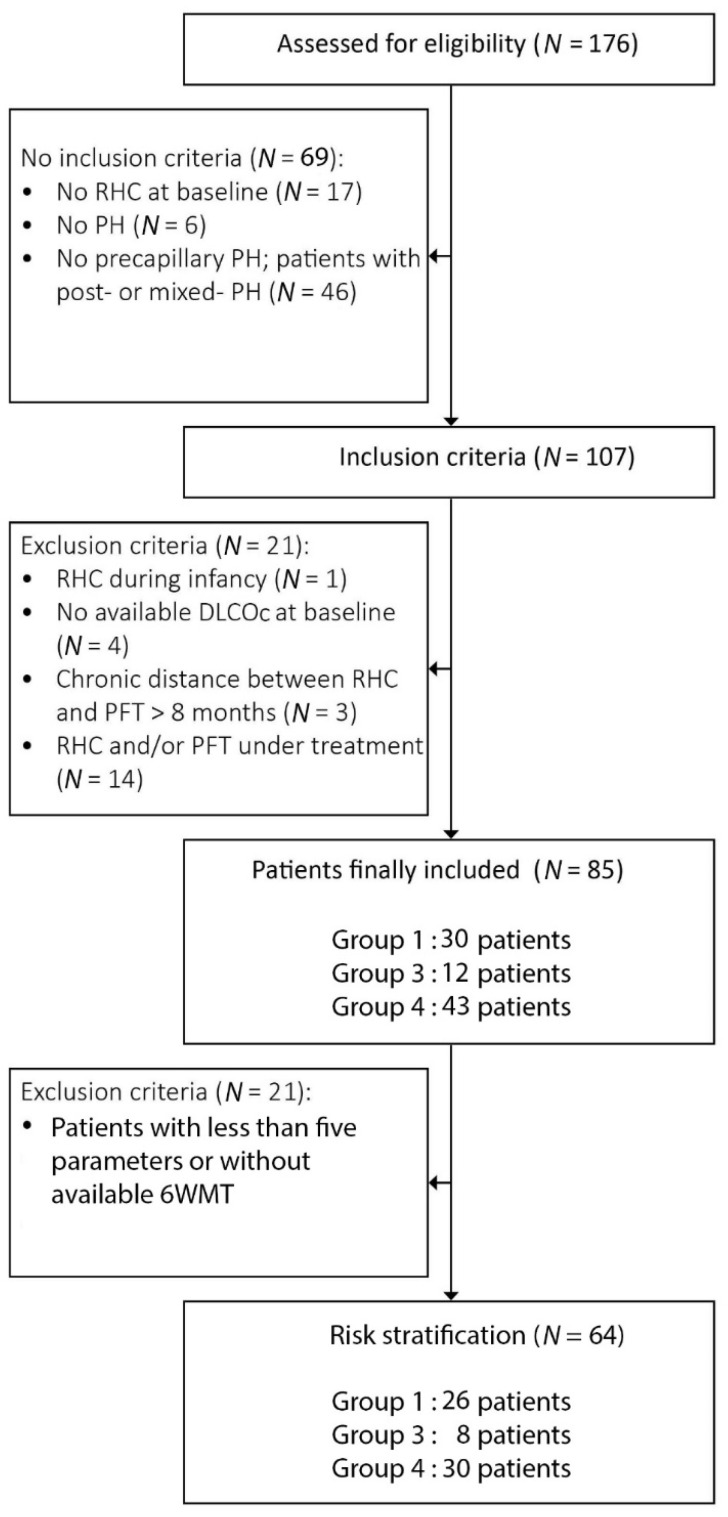
Flow chart of inclusion and exclusion criteria of the study. Abbreviations: RHC, right heart catheterisation; PH, pulmonary hypertension; PFT, pulmonary functional testing; DLCOc, carbon monoxide diffusing capacity.

The study population included 85 patients. Figure 1 illustrates the flow chart of inclusion and exclusion criteria. Overall, 30 patients were classified into Group 1, 12 into Group 3 and 43 into Group 4. Median age (range) at diagnosis was 63 (23–89) years and 55% (47/85) were females. In total, 52 (59%) patients exhibited dyspnea class NYHA III-IV. Median DLCOc was 61 (13–102)%, while median DLCOc was 56.5 (28–83)% for patients in diagnostic group 1, 38 (13–59)% in group 3 and 69 (38–102)% in group 4. Median mPAP was 43 (27–88) mmHg, sPAP was 72 (35–146) mmHg, CI was 2.5 (1.4–6) L/min·m^2^, and PVR was 7.6WU (3.1–20). Median distance in 6MWT was 355 (120–705) m. Median follow-up time was 3.67 (0.01–12.32) years. Overall, 13 (15%) patients died and 2 (2%) underwent lung transplantation within a median time interval of 2.6 (0.9–8.5) years from diagnosis. Events occurred in each diagnostic group as following: in group 1, three (3%) patients died and one (1%) underwent lung transplantation; in group 3, five (5%) patients died and in group 4, five (5%) patients died and one (1%) was lung transplanted. In total, 32 (38%) transplant-free survivals were followed-up for >5 years.

### 3.1. DLCO and Variables of the COMPERA Score

Overall, 32 patients presented with DLCOc < 55% and 53 with DLCOc ≥ 55%. Distribution of patients’ functional status parameters according to DLCOc level are shown in Table 1. Patients with DLCOc < 55% displayed higher-grade dyspnea (*p* = 0.005) and shorter 6MWT distance (*p* = 0.015).

**Figure 2:**
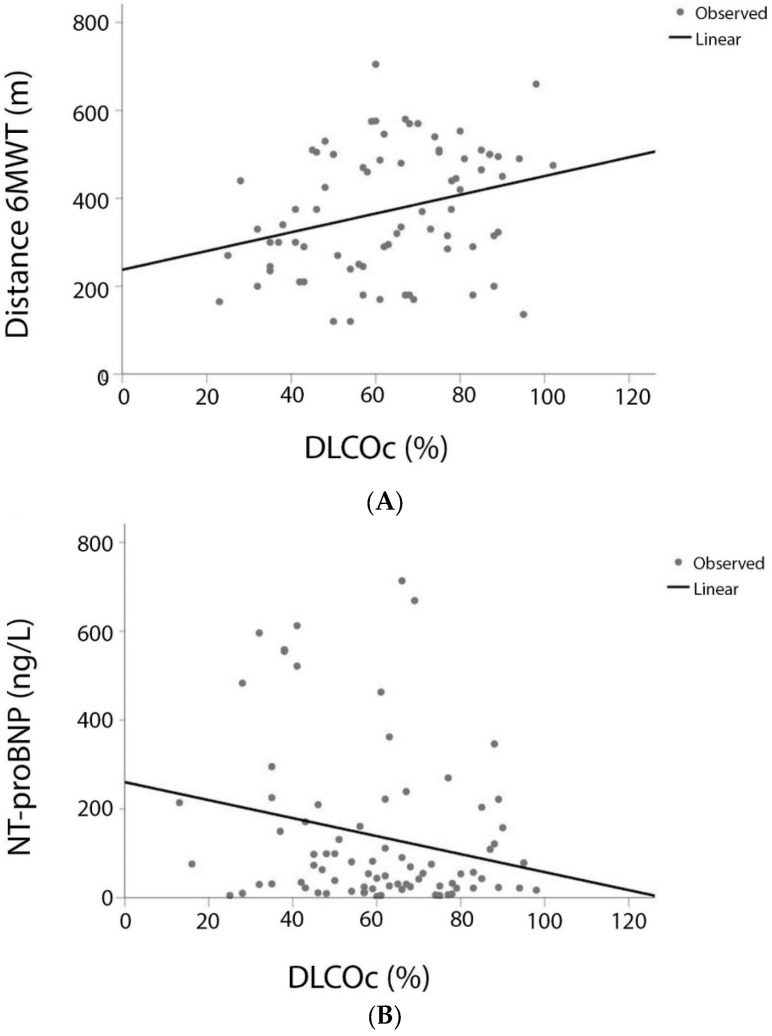
Correlation between (**A**). DLCOc and 6MWT distance (r = 0.288, *p* = 0.013) and (**B**). DLCOc and NT-proBNP (r = −0.222, *p* = 0.048). Abbreviations: DLCOc, carbon monoxide diffusing capacity; 6MWT, 6-min walk test; NT-proBNP, N-terminal pro-brain natriuretic peptide.

DLCOc was positively correlated with 6MWT distance (r = 0.288, *p* = 0.013), weakly negatively correlated with NT-proBNP (r = −0.222, *p* = 0.048) (Figure 2), whereas it was not at all correlated with mPAP (r = −0.002, *p* = 0.987), PVR (r = −0.115, *p* = 0.313), RAP (r = −0.210, *p* = 0.076), and CI (r = 0.082, *p* = 0.479). As expected, DLCOc levels were significantly lower in diagnostic Group 3 compared to diagnostic Group 1 or 4 patients (*p* < 0.001). Nevertheless, after adjusting for age, gender, and diagnostic group, DLCOc was still significantly correlated with 6MWT distance (r = 0.552, *p* = 0.031), but no more with NT-proBNP (r = 0.234, *p* = 0.096).

### 3.2. DLCO and Transplant-Free Survival

**Figure 3:**
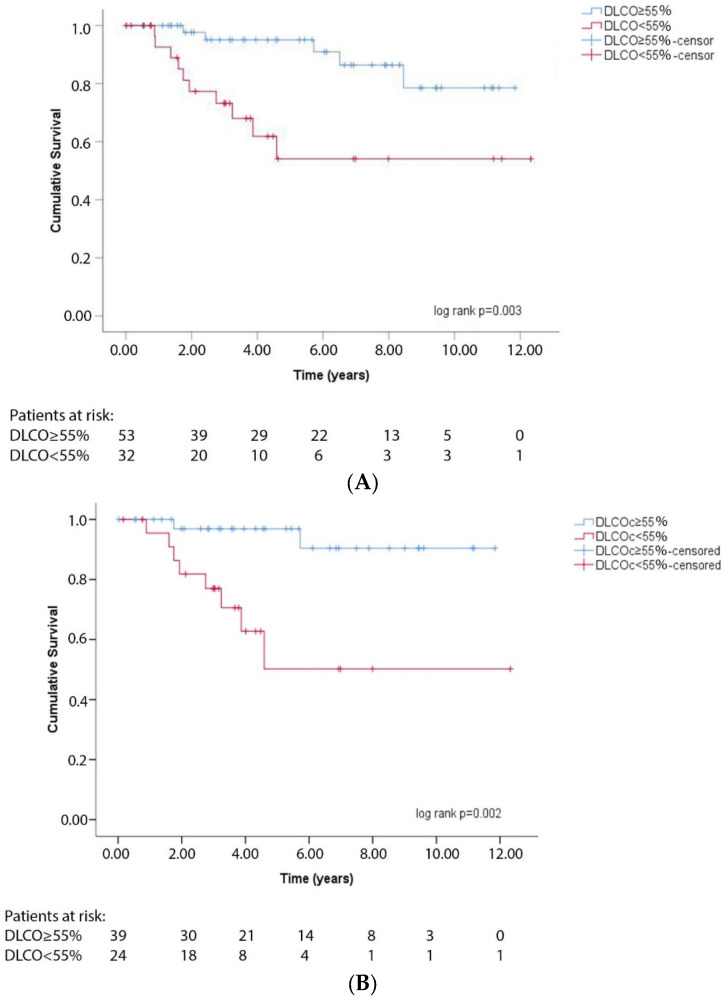
Kaplan–Meier analysis for DLCOc < 55% and transplant-free survival (**A**). in all patients (N = 85) (log rank *p* = 0.002) and (**B**). in intermediate and high-risk category patients (*N* = 63) (log-rank *p* = 0.002).

Following Cox regression analysis, DLCOc was identified as a negative transplant-free survival risk factor (HR 0.939, 95% CI: 0.908–0.971, *p* < 0.001), even after adjusting for diagnostic group, NT-proBNP, 6MWT distance, and CI (HR 0.932, 95% CI: 0.882–0.986, *p* = 0.013) (Table 2). Moreover, DLCOc was negatively associated with 3- and 5-year transplant-free survival rates (OR 0.932, 95% CI: 0.886–0.980, *p* = 0.006 and OR 0.874, 95% CI: 0.802–0.953, *p* = 0.002, respectively). Furthermore, Kaplan–Meier analysis revealed that a cut-off value of 55% for DLCOc exhibited the best discrimination between transplant-free survival and deaths for the whole follow-up period (log rank *p* = 0.002) (Figure 3A), even after adjusting for diagnostic group, NT-proBNP, 6MWT distance, and CI (HR 5.706, 95% CI: 1.203–27.056, *p* = 0.028). Of note, DLCOc < 55% was associated with a reduced transplant-free survival rate at both 3- and 5-year follow-up (OR 7.000, 95% CI: 1.310–37.403, *p* = 0.023 and OR 21.667, 95% CI: 3.733–125.767, *p* = 0.001, respectively).

### 3.3. DLCO and Risk Stratification

Risk stratification assessment according to the COMPERA model was performed in 64 patients; of these, 26 were classified in PH Group 1, 8 in Group 3 and 30 in Group 4. Events occurred in each diagnostic group as follows: in group 1, three (4%) patients died and one (1%) underwent lung transplantation; in group 3, three (4%) patients died and in group 4, two (3%) patients died and one (1%) was lung transplanted. In total, one patient was classified as low-risk, 49 (77%) as intermediate-risk, and 14 (22%) as high-risk categories. During the follow-up, eight patients died, five of whom were classified in the intermediate-risk, while two high-risk patients were transplanted. Eight (57%) patients in the high-risk category, sixteen (33%) patients in the intermediate-risk, and no patient in the low-risk category exhibited DLCOc < 55%.

Given the limited number of low-risk group patients, further analyses were performed only in patients included in intermediate and high-risk categories. DLCOc was identified as a significant risk factor for transplant-free survival after adjusting for risk stratification category (HR 0.934, 95% CI 0.890–0.980, *p* = 0.005) (Table 2). Furthermore, DLCOc < 55% was significantly associated with worse transplant-free survival prognosis (log-rank *p* = 0.002) (Figure 3B), even after adjusting for risk stratification category (HR 7.082, 95% CI: 1.449–34.604, *p* = 0.016) (Table 2).

Based on DLCOc first (46%) and third (77%) quartiles, patients with DLCOc values ≥ 75% were included in the low-risk category, those with DLCOc values ≥ 45 and <75% in the intermediate-risk category, and those with DLCOc values < 45% in the high-risk category. We then calculated a modified risk assessment after DLCOc inclusion in the COMPERA score. As a result, 46 patients were classified as modified intermediate-risk and 17 as modified high-risk categories. Compared to the original COMPERA risk stratification, three patients from the intermediate-risk category moved to the high-risk category. As expected, the association between COMPERA’s original risk-category and transplant-free survival was statistically significant (HR 3.78, 95% CI: 1.094–13.064, *p* = 0.036). The association between the modified-risk category and transplant-free survival remained also statistically significant (HR 4.60, 95% CI: 1.294–16.352, *p* = 0.018). The Akaike information criterion (AIC) of Cox regression analysis based on the original COMPERA risk stratification was 71.402, whereas it was 69.924 for the modified-risk stratification. Therefore, the AIC of the Cox regression analysis based on the modified-risk stratification model is lower compared to the original COMPERA risk model and fits better the results of our study. 

**Figure 4:**
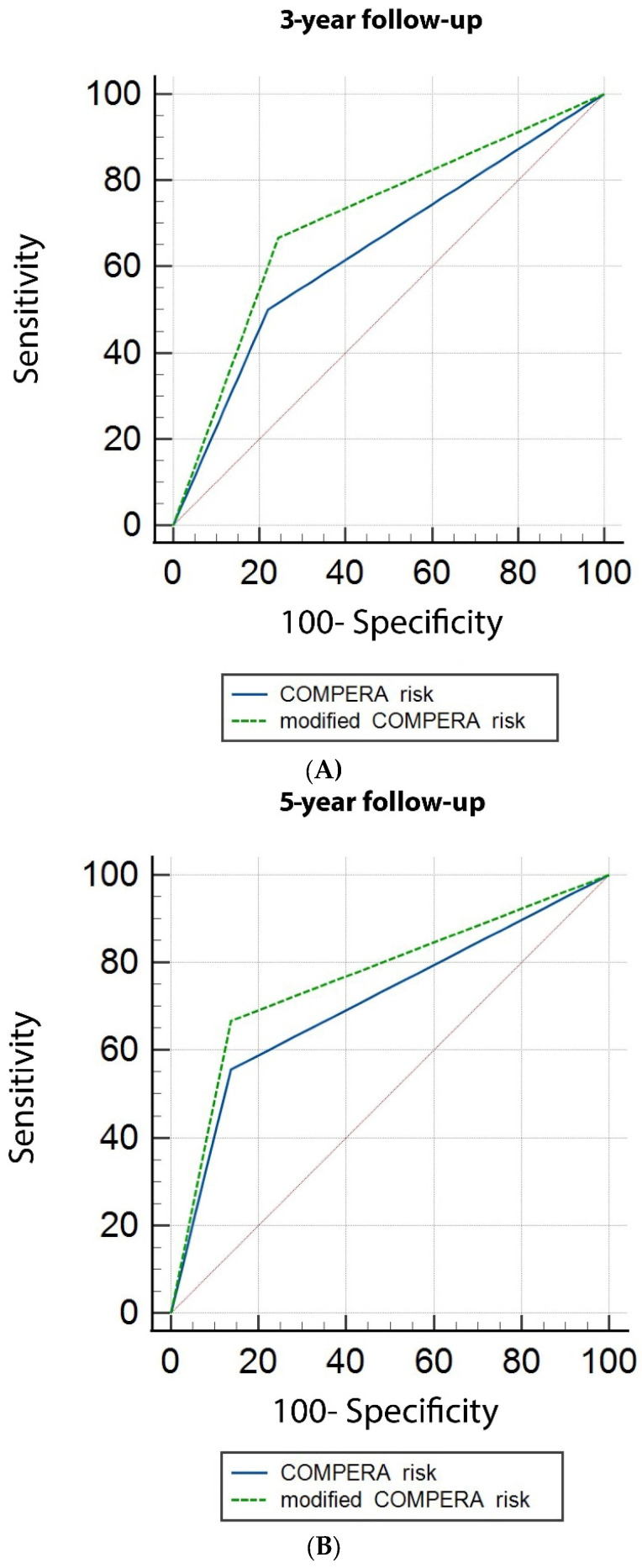
Area under the curve (AUC) for transplant-free survival probability over 3- and 5-year follow-up, in intermediate- and high-risk category patients, using the COMPERA and the modified-risk stratification strategy. (**A**). Five-year transplant-free survival probability with the COMPERA risk: AUC 0.640, Standard error (SE) 0.116, 95% confident interval (CI) 0.487–0.775 and with the modified-risk stratification strategy: AUC 0.711, SE 0.111, 95% CI 0.561–0.834. (**B**). five-year transplant-free survival probability with the COMPERA risk: AUC 0.710, SE 0.096, 95% CI 0.520–0.858 and with the modified-risk stratification strategy: AUC 0.760, SE 0.091, 95% CI 0.579–0.898.

The area under the curve (AUC) from the ROC curves, for predicting three-year transplant-free survival in intermediate and high-risk category patients by the COMPERA risk assessment, was 0.640, standard error (SE) 0.116, 95% CI 0.487–0.775 and by the modified-risk stratification strategy 0.711, SE 0.111, 95% CI 0.561–0.834. For predicting five-year transplant-free survival by COMPERA risk, the AUC was 0.710, SE 0.096, 95% CI 0.520–0.858 and by the modified-risk stratification strategy, the AUC was 0.760, SE 0.091, 95% CI 0.579–0.898 (Figure 4). However, the difference between the AUC did not reach statistical significance (at 3- and 5-year follow-up respectively: Δ AUC 0.071, SE 0.084, *p* = 0.398 and Δ AUC 0.056, SE 0.056, *p* = 0.317).

**Figure 5:**
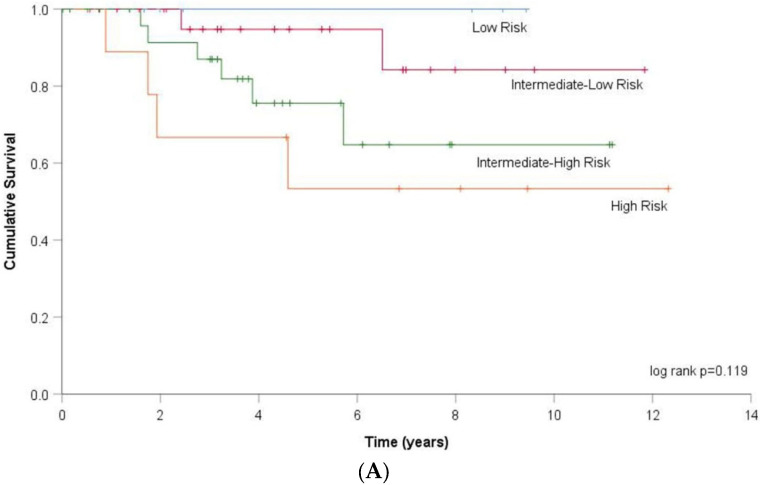

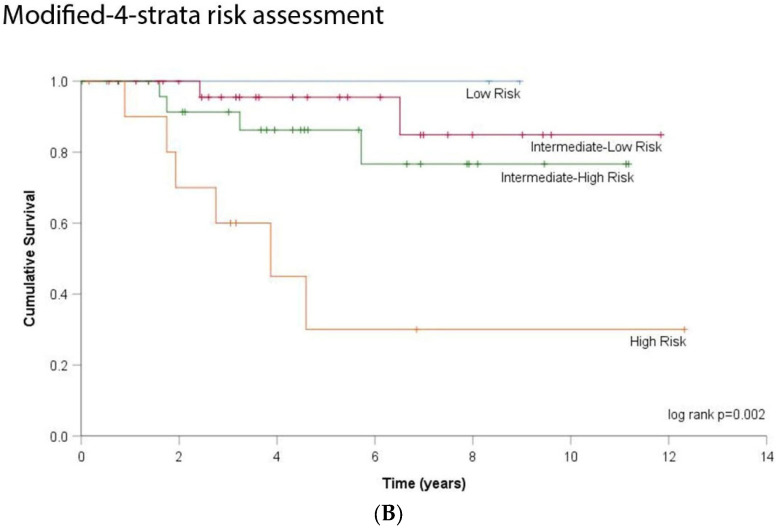
Kaplan–Meier analysis for transplant-free survival and (**A**). 4-strata risk assessment model (log rank *p* = 0.119) and (**B**). modified-4-strata risk assessment model (log rank *p* = 0.002).

We, then, calculated the 4-strata risk assessment on 69 patients according to COMPERA 2.0. In total, 7 (10%) patients were classified as low-risk, 25 (36%) as intermediate-low risk, 28 (41%) as intermediate-high risk and 9 (13%) as high-risk categories. Based on DLCOc first (46%), median (61%) and third (77%) quartiles, patients with DLCOc values ≥ 75% were included in the low-risk category, those with >60% and <75% in the intermediate-low risk category, those with ≥45% and ≤60% in the intermediate-high risk category and those with DLCOc values < 45% in the high-risk category. After DLCOc inclusion in the COMPERA 2.0 score, the modified 4-strata risk assessment was calculated. Compared to the 4-strata risk assessment, 15 patients changed risk category. Three patients moved from low-risk to intermediate-low risk category, three patients moved from intermediate-low to intermediate-high risk category, five patients moved from intermediate-high to high-risk category, whereas three patients moved from high to intermediate-high risk category and one patient from intermediate-high to intermediate-low risk category. The association between 4-strata risk assessment model and transplant-free survival did not meet statistical significance by Kaplan–Meier analysis (log rank *p* = 0.119). However, the association between the modified-4-strata risk category and transplant-free survival reached statistical significance (log-rank *p* = 0.002) (Figure 5). In Cox regression analysis, transplant-free survival was significantly associated with both 4-strata risk assessment (HR 2.331, 95% CI 1.135–4.787) and modified-4-strata risk assessment (HR 3.524, 95% CI 1.568–7.919, *p* = 0.002). The AIC of Cox regression analysis based on the 4-strata COMPERA 2.0 risk stratification was 85.725, whereas it was 80.952 for the DLCO modified-4-strata risk stratification, showing therefore a better fit for the latter.

## 4. Discussion

The current study involving PH patients from 3 diagnostic groups clearly showed that: (1) DLCOc was at best loosely correlated with established prognostic parameters, such as the distance of the 6MWT and NT-proBNP levels, providing incremental independent information; (2) Baseline DLCOc and transplant-free survival were strongly related, independently of diagnostic group, NT-proBNP, 6MWT distance, and CI; (3) DLCOc < 55% identified a sub-group of 32 patients with more severe dyspnea, shorter walking distance, more hypoxemic during exercise, and worse transplant-free survival; 4) Integrating DLCOc in the COMPERA risk assessment-score provided statistically significant prediction of transplant-free survival. This modified-risk stratification model is therefore a promising derivation model but should be externally validated before larger clinical use.

DLCO’s contribution to survival prediction, as highlighted in our study, is consistent with previously published literature data. Low DLCO levels were independently associated with poor survival in PH patients with connective-tissue disease [27,28]. Similarly, in a cohort of 493 patients, DLCO was recently proved to be an independent predictor of higher mortality in idiopathic PAH patients; specifically DLCO <45% was associated with significantly lower 1- and 5-year survival rates [29]. In an earlier study, PAH patients with DLCO < 43% exhibited poor survival, irrespective of functional class, lung function abnormalities, and hemodynamic variable variations [16]. Furthermore, concerning PH group 3, Rose et al. demonstrated that, among several parameters analyzed, DLCO was the only independent predictor of mortality [30]. Moreover, in patients with chronic obstructive pulmonary disease, for every percent predicted decrease in DLCO, mortality rates increased by 4% [31]. For PH group 4, DLCO was considered to be a determinant survival risk factor in medically-treated patients [32,33]. Finally, in a recent prospective study, including patients with not only precapillary but also postcapillary PH, DLCO was the strongest predictor of mortality in all population and etiological subgroups, except in group 4 [34]. Therefore, DLCO appears to be a significant parameter, irrespective of the PH diagnostic group.

Surprisingly, DLCO cut-off values for predicting survival are quite different among published studies, ranging from 32% to 60% [27,29,30,34]. Two larger studies have identified a DLCO cut-off at 45% for idiopathic PAH [17,29]. Moreover, in the REVEAL 2.0 score, DLCO < 40% value was defined to increase the score by one point. In our study, we have demonstrated that, even at a higher DLCOc level, notably 55%, such a parameter is associated with poorer clinical performance and worse prognosis. Therefore, a threshold of DLCO < 55% may already be taken into consideration in the everyday clinical practice.

The discrepancy of DLCO cut-offs across different studies may be explained by several causes; it may thus be the variable proportion of patients with systemic-sclerosis associated PH, as this latter population has been shown to display significantly lower DLCO levels than patients with idiopathic PAH [28]. Another explanation of further DLCO decreases may be the presence of patients with pulmonary veno-occlusive disease, exhibiting rather indistinguishable hemodynamics from PAH. Smoking is also thought to be a cause of reducing DLCO in PAH patients, even in the absence of any obvious emphysema or interstitial lung disease upon chest imaging [29], leading to the relatively new concept of smoking-related vasculopathy [35]. Finally, a vanishing pulmonary capillary syndrome as physio-pathological cause of some PH forms has also been proposed [36]. Larger future studies for each diagnostic subcategory are thus warranted to better specify such DLCO cut-off levels.

Distance achieved during the 6MWT is a well-known surrogate marker of outcome, having been used as a primary endpoint in numerous clinical trials. A 6MWT distance >390 m was shown to predict survival in all etiologic groups of the Giessen PH Registry. In this registry, however, the DLCO values were not investigated [37]. In addition, in the COMPERA cohort, a significant low 6MWT (<165 m) or marked 6MWT deterioration (>70 m, >15% reduction from baseline) was indicative for a high-risk of clinical deterioration [38]. In our study, we demonstrated that DLCOc was accounting for a very small part of 6MWT distance variance in pre-capillary PH. According to our hypothesis, the many mechanisms involved in DLCO impairment, including ventilation-perfusion mismatch, hypoxic vasoconstriction, and right heart dysfunction with decreased cardiac output, can altogether be the cause of exercise limitation. However, a recent study involving patients from all five PH groups was unable to find any relation between DLCO values and walking distance [39].

NT-proBNP may be elevated in treatment-naive PH patients, which is associated with disease severity and right ventricle enlargement [40]. We demonstrated a weak inverse relation between DLCOc and NT-proBNP in the initial evaluation of PH patients, already described during follow-up of patients with idiopathic PAH [41]. From a pathophysiological perspective, NT-proBNP levels are associated with heart disease, resulting from an abnormal end-diastolic wall stress either in the left or right ventricle. In precapillary PH patients with right ventricular dysfunction, serial measurements allow for monitoring and guiding treatment [42]. The inverse relation between NT-proBNP and DLCO can probably be seen as two simultaneous consequences of the pulmonary vascular disease with alveolo-capillary membrane’s alteration.

Despite the strong evidence that DLCO predicts survival, the current 2015 ESC/ERS guidelines along with those of the 2018 World Symposium on PH do not include DLCO in the risk assessment algorithm [43]. One reason could be that DLCO, contrary to either NYHA functional class or the 6MWT, is not considered to be a modifiable parameter. In other words, what would be the benefit for the clinician to take into account a surrogate marker of disease severity that could not be improved by therapy? However, robust prospective studies on PH treatments’ impact on DLCO level are still lacking. More importantly, whether or not DLCO levels can ultimately be modified by PH therapy does not necessarily dismiss its prognostic value. We can even go a step further. Notably, we can question the efficacy of current PH medications unable to correct the altered physiology of the pulmonary vasculature. In our study, after incorporating DLCO in the 2015 risk stratification model, we demonstrated that lower DLCOc levels strongly influenced transplant-free survival rates, independently of standard risk stratification. Furthermore, adding DLCO to recently published 4-strata risk assessment model can significantly predict transplant-free survival. As observed in Figure 5, the high-risk category had clearly a more dramatic attrition compared to the others. The discrimination between high-intermediate and low-intermediate risk, however, was not observed. Another key study finding has been that the AIC of Cox regression analysis based not only on the modified-COMPERA risk stratification but also on the modified-4-strata risk stratification, better fit our results compared to that based on the original stratification. Our study has, thus, highlighted DLCO’s significance in the evaluation of patient prognosis. This suggests that including DLCO into patient risk stratification scores may enable further identification of high-risk patients by noninvasive methods, along with a more individualized and personalized treatment. Some caution is warranted, however, as the differences in the ROC curves for predicting survival at three and five years are not statistically different between the two models. Other non-invasive parameters are also eligible for the assessment of prognosis in PH as shown for echocardiography [44]. As risk stratification has been recently shown to be critical in the initial choice of treatment, we think that any further improvement in prognosis prediction deserves consideration [45].

This study displays several limitations. First of all, a single-center retrospective long-term cohort design was employed. Second, the final number of patients in each diagnostic category was relatively small, and the absence of any statistically significant correlations between DLCO and clinical risk category may thus have resulted from a beta error. Third, although the 2015 risk stratification model has been mainly approved for PAH Group 1 patients, and, as the majority of registries exclude PH patients from other groups [46], we have used the current 2015 ESC/ERS risk assessment strategy by analogy. Such an extension strategy has, however, recently be applied in order to evaluate prognosis in CTEPH [47,48] and in interstitial lung disease patients [49]. Finally, invasive procedures such as pulmonary endarterectomy [50] and balloon angioplasty are specific treatments known to improve the survival of CTEPH patients and may contribute to the better prognosis of this PH group compared to group 1 and 3. In contrast to lung transplantation, we did not include pulmonary endarterectomy as a censoring event in the survival curves, considering it as a standard of care for eligible patients with CTEPH. A proportion of elderly patients may have refused a pulmonary endarterectomy, though technically operable, which also impacts survival [51]. Balloon angioplasty was introduced in our centre only since 2017 and therefore had marginal impact in the present study.

## 5. Conclusions

We demonstrated that lower DLCO levels are strongly associated with worse overall transplant-free survival in precapillary PH, independently of the 2015 risk stratification score. In view of these results, DLCO may be considered at the initial PH risk assessment. DLCO’s inclusion into current risk evaluation algorithm could be promising but needs further evaluation and validation. Larger prospective trials in defined PH groups are warranted to support this observation and to assess whether successful PH therapy may eventually improve DLCO levels.

## Figures and Tables

**Table 1:** Distribution of patients’ functional status parameters according to DLCOc level. Abbreviations: DLCOc, carbon monoxide diffusion capacity; NYHA, New York Heart Association; Va, alveolar volume, KCO, transfer coefficient for carbon monoxide; FEV1, forced expiratory volume in one second; mPAP, mean pulmonary arterial pressure; sPAP, systolic pulmonary arterial pressure; CI, cardiac index; mRAP, mean right atrial pressure, PAWP, pulmonary arterial wedge pressure; 6MWT, 6-min walk test; min SpO2, minimum peripheral capillary hemoglobin oxygen saturation measured during effort; NT-proBNP, N-terminal pro-brain natriuretic peptide. Significant *p* values are highlighted in bold.

	Data (*N*, %)	DLCOc < 55% *N* = 32	DLCOc ≥ 55% *N* = 53	*p*
Clinical characteristics				
Age at diagnosis (range)	85 (100)	65 (32–89)	61 (23–89)	0.196
Female sex (*N* = 47, 53%)		17 (53%)	30 (57%)	0.824
Dyspnea NYHA III-IV (*N*, %)	82 (96)	25 (78)	27 (51)	**0.005**
Smoking status (*N*, %)	84 (99)			
Never smokers (*N*, %)		11 (34)	25 (47)	0.364
Pulmonary function				
Median DLCOc (%) (range)	85 (100)	41.5 (13–54)	71 (56–102)	
Median Va (%) (range)	79 (93)	72 (41–100)	88 (64–124)	**<0.001**
Median KCO (%) (range)	83 (98)	59 (18–102)	85 (50–122)	**<0.001**
Median FEV1 (%) (range)	83 (98)	71 (22–128)	92.5 (37–142)	**<0.001**
Haemodynamics				
mPAP (mmHg) (range)	85 (100)	44 (28–88)	42 (27–83)	0.552
sPAP (mmHg) (range)	83 (98)	69 (41–142)	75 (35–146)	0.437
CI (L/min/m^2^) (range)	76 (89)	2.48 (1.4–3.39)	2.5 (1.45–6)	0.365
mRAP (mmHg) (range)	72 (85)	9 (1–18)	6 (0–16)	**0.042**
PAWP (mmHg) (range)	79 (93)	11 (6–15)	10 (2–15)	0.150
6MWT				
Distance (m) (range)	74 (87.5)	300 (120–530)	445 (136–705)	**0.015**
Resting SpO2 (%) (range)	74 (87.5)	93 (65–98)	94 (83–98)	0.542
Min SpO2 (%) (range)	75 (88)	83 (44–100)	87 (68–96)	**0.017**
Serum biomarkers				
NT-proBNP (ng/L) (range)	80 (94)	978 (52–6127)	466.5 (32–7137)	0.072

**Table 2:** Association between DLCOc and survival. * Adjustment for diagnostic group, NT-proBNP, 6MWT distance and CI. ** adjustment for risk category. Abbreviations: DLCO, carbon monoxide diffusion capacity; NT-proBNP, N-terminal pro-brain natriuretic peptide; 6MWT, 6-min walk test; CI, cardiac index. Significant *p* values are highlighted in bold.

	Survival					
	All Patients (*N* = 85)					
	Univariate Analysis			Multivariate Analysis *		
	HR	95% CI	*p*-Value	HR	95% CI	*p*-Value
DLCOc	0.939	0.908–0.971	**<0.001**	0.932	0.882–0.986	**0.013**
DLCOc < 55%	4.676	1.583–13.817	**0.005**	5.665	1.196–26.842	**0.029**
	Patients in intermediate- and high-risk category (*N* = 63)					
	Univariate analysis			Multivariate analysis **		
DLCOc	0.931	0.890–0.975	**0.002**	0.934	0.890–0.980	**0.005**
DLCOc < 55%	8.407	1.755–40.261	**0.008**	7.082	1.449–34.604	**0.016**

## Data Availability

The original medical data that support the findings of this study are not publicly available because this information could compromise the privacy of the patients. However, anonymised raw data are available upon request from the first author.
